# Balance ability in 7- and 10-year-old children: associations with prenatal lead and cadmium exposure and with blood lead levels in childhood in a prospective birth cohort study

**DOI:** 10.1136/bmjopen-2015-009635

**Published:** 2015-12-30

**Authors:** Caroline M Taylor, Rachel Humphriss, Amanda Hall, Jean Golding, Alan M Emond

**Affiliations:** 1 Centre for Child and Adolescent Health, School of Social and Community Medicine, University of Bristol, Bristol, UK; 2 School of Social and Community Medicine, University of Bristol, Bristol, UK; 3 Children's Hearing Centre, University Bristol NHS Foundation Trust, Bristol, UK

**Keywords:** Lead, Cadmium, Vestibular function, Balance, Pregnancy, ALSPAC

## Abstract

**Objectives:**

Most studies reporting evidence of adverse effects of lead and cadmium on the ability to balance have been conducted in high-exposure groups or have included adults. The effects of prenatal exposure have not been well studied, nor have the effects in children been directly studied. The aim of the study was to identify the associations of lead (in utero and in childhood) and cadmium (in utero) exposure with the ability to balance in children aged 7 and 10 years.

**Design:**

Prospective birth cohort study.

**Participants:**

Maternal blood lead (n=4285) and cadmium (n=4286) levels were measured by inductively coupled plasma mass spectrometry in women enrolled in the Avon Longitudinal Study of Parents and Children (ALSPAC) during pregnancy. Child lead levels were measured in a subsample of 582 of ALSPAC children at age 30 months.

**Main outcome measures:**

Children completed a heel-to-toe walking test at 7 years. At 10 years, the children underwent clinical tests of static and dynamic balance. Statistical analysis using SPSS V.19 included logistic regression modelling, comparing categories of ≥5 vs <5 µg/dL for lead, and ≥1 vs <1 µg/L for cadmium.

**Results:**

Balance at age 7 years was not associated with elevated in utero lead or cadmium exposure (adjusted OR for balance dysfunction: Pb 1.01 (95% CI 0.95 to 1.01), n=1732; Cd 0.95 (0.77 to 1.20), n=1734), or with elevated child blood lead level at age 30 months (adjusted OR 0.98 (0.92 to 1.05), n=354). Similarly, neither measures of static nor dynamic balance at age 10 years were associated with in utero lead or cadmium exposure, or child lead level.

**Conclusions:**

These findings do not provide any evidence of an association of prenatal exposure to lead or cadmium, or lead levels in childhood, on balance ability in children. Confirmation in other cohorts is needed.

**Strengths and limitations of this study**

- Data were collected prospectively in a population-based study.
- The number of participants was large compared with several comparable studies.
- Measures of Pb and Cd do not necessarily reflect lifetime exposure.
- Balance measures have a poor test–retest reliability.

## Introduction

Balance, or postural stability, is defined as the ability to keep the centre of gravity over the base of support.^1^ The maintenance of balance underpins the ability to carry out nearly all daily activities. Balance impairment in adults is also a major cause of falls and of fall-related injuries, such as hip fracture, which can cause isolation and make it difficult to live independently. The control of balance is complex and is dependent on sensory inputs from the vestibular and visual systems, neural processing centres in the central nervous system, and motor inputs from the proprioceptive centre. Functional damage or deficits in any of these systems can lead to balance dysfunction, which can be associated with low self-esteem, anxiety and loss of confidence in children.^2^

Lead and cadmium are toxic metals: the effects of lead on neurocognitive and behavioural functions in children are well documented,^3–5^ but those of cadmium are less clear.^6–8^ Lead passes freely through the placenta, so that the ratio of fetal to maternal blood lead is about 0.8, although the placenta can act as a partial barrier to cadmium.^9^ The fetus is particularly vulnerable to the effects of these metals because of high rates of cell division and development. The development of the inner ear and vestibular function spans the whole of the period of gestation (eg, the membranous labyrinth is complete by week 7 with development of the bony labyrinth from weeks 9 to 23; the vestibular apparatus is in an adult-like form by week 25, and is active by week 32; vestibular ganglions develop from week 12 and reach maturity at week 39, etc^10^). Thus, prenatal exposure to lead and cadmium may have adverse effects on the development of the inner ear, and hence, on vestibular function and balance ability in later childhood.

It was noted in the 1980s that children who survived acute lead encephalopathy had ataxia and experienced difficulties in maintaining postural balance.^11^ This led to a series of studies in children with somewhat more moderate levels of lead exposure (5.0–20.7 µg/dL) showing that the child's lead level was associated with balance dysfunction and sway oscillation.^12–16^ To the best of our knowledge, there are no reports of the effect of cadmium on balance ability in children. However, a recent study of lead and cadmium levels in adults in the US National Health and Nutrition Examination Survey found preliminary evidence of an association of lead and cadmium with balance and vestibular function.^17^ In addition, altered postural balance response has been reported in adult workers occupationally exposed to lead^18–20^ and cadmium.^21^ These results require confirmation in other cohorts and particularly in children.

The aims of our study were to investigate the associations of in utero exposure to lead and cadmium, and lead levels in children, and on balance in childhood using data obtained from the Avon Longitudinal Study of Parents and Children (ALSPAC).

## Methods

We first modelled associations of in utero exposure to lead and cadmium, using maternal blood levels during pregnancy, with clinical measures of balance (dynamic and static) at 7 and 10 years of age. We also investigated associations with questionnaire items related to balance repeated at 30, 42 and 81 months, and further items at 10 years. We also modelled the associations of child levels of lead with the balance variables.

### The ALSPAC study

The study sample was derived from the ALSPAC study, a population-based study investigating environmental and genetic influences on the health, behaviour and development of children. All pregnant women in the former Avon Health Authority with an expected delivery date between 1 April 1991 and 31 December 1992 were eligible for the study; 14 541 pregnant women were initially enrolled, resulting in a cohort of 14 062 live births.^22^ The social and demographic characteristics of this cohort were similar to those found in UK national census surveys.^23^ Further details of ALSPAC are available at http://www.bris.ac.uk/alspac.

### Collection, storage and analysis of blood samples

#### Maternal blood samples

Whole blood samples were collected in trace-element-free vacutainers (Becton and Dickinson, Oxford, UK) by midwives as early as possible in pregnancy. The median gestational age at the time of blood sampling was 11 weeks. The IQR was 9–13 weeks, and 93% of the samples were collected at <18 weeks of gestation. Whole blood samples were stored in the original tube at 4°C at the collection site before being transferred to the central Bristol laboratory within 1–4 days. Samples were at ambient temperature during transfer (up to 3 h). They were then stored at 4°C until analysis. Samples were analysed for lead and cadmium using inductively coupled plasma mass spectrometry in standard mode by R Jones at the Centers for Disease Control and Prevention (CDC), Bethesda, Maryland, USA (CDC Method 3009.1). Quality control was monitored as outlined in Golding *et al*.^24^ The analyses were completed on 4285 samples for lead and 4286 for cadmium. One sample had a lead level below the limit of detection (0.29 µg/dL); 1119 samples were below the lower limit of detection for cadmium (0.20 µg/L). These samples were assigned a value of 0.7 times the lower limit of detection (LOD/√2).^25^ ^26^

#### Child blood samples

Details of the selection of the subsample of children and analysis of the blood samples have been reported previously in detail.^3^ ^27^ In brief, a 10% randomly selected subsample of parents whose babies were born in the last 6 months of the ALSPAC study were invited to attend a research clinic (Children in Focus, CIF). At age 30 months, parental consent was sought for a venous blood sample, and was given by 81% of the 1135 children in the CIF group. The sample was drawn into lead-free tubes from 653 (71%) of children attending the clinic. However, 69 samples were insufficient, leaving 582 samples for analysis. Analysis was by atomic absorption spectrometry (Southampton General Hospital, UK) with appropriate quality controls.

### Balance variables

#### Clinic measures

Full details of the balance outcomes including details of the measurements and validity have previously been published.^28^ In brief, at age 7 years, the heel-to-toe walking test of the Movement Assessment Battery for Children^29^ was conducted with the total number of successful steps out of a maximum of 15 recorded. At age 10 years, a range of tests were used to assess balance: (1) walking along a beam, heel-to-toe, eyes open; (2) heel-to-toe balance on a beam, eyes closed; and (3) standing on one leg, eyes closed. Each child had two attempts at beam walking; for tests of static balance, children only had a second attempt if they failed to achieve the maximum score on the first attempt.^28^ These tests were based on standard clinical tests to assess balance in children, and have significant commonality with the balance subtest of both editions of the Bruininks–Oseretsky Test of Motor Proficiency.^30^ ^31^ The measures are also in common use when testing balance informally in the paediatric clinic.

#### Questionnaire items

The primary caregiver (usually the mother) received a series of postal self-completion questionnaires. The questionnaires are available from the study website (http://www.bris.ac.uk/alspac/researchers/data-access/data-dictionary/). When their child was aged 18, 30, 42 and 81 months, the parent completing the questionnaire was asked to indicate ‘Yes, can do well’/‘Has only done once or twice’/‘Has not yet started’, in response to the statement ‘He/She can balance on one foot for at least 1 s’. When their child was aged 10 years, the parent was asked to indicate ‘Very well’/‘Just OK’/‘Can almost’/‘Not at all’ in response to the following questions: How well can your child stand on one leg in a stable position (eg, when putting on trousers, skirt)?; How well can your child ride a bike (without stabilisers)?; How well can your child walk in the dark?

### Confounding variables

Information on passive smoking exposure during the week and at weekends was obtained from questionnaires at 77 and 103 months. Information on traffic levels, type of accommodation, lowest level of accommodation and maternal education were obtained from questionnaires completed by the mother during pregnancy. Dietary calcium and iron intakes at 7 and 10 years were derived from food frequency questionnaires as previously described in detail.^32^

### Statistical analysis

Statistical analysis was carried out with IBM SPSS Statistics V.21. Balance measures were derived as previously described.^28^ In brief, for the heel-to-toe test at age 7 years, the number of steps (maximum 15) was categorised into 0–5, 6–10 and 11–15 steps for categorical associations, and 1–14 (failed to complete in 20 s) versus 15 steps (successfully completed in 20 s) for regression analyses. For the measure of dynamic balance at 10 years (beam walking test) the mean of two attempts was categorised into quartiles. For measures of static balance at age 10 years (heel-to-toe balance on a beam with eyes closed/standing on one leg eyes closed), the sum of the score (s) from both attempts was calculated. Children who scored the maximum of 20 on the first attempt and so did not have a second attempt were given a final score of 40. The final scores were put into four categories (0–9, 10–19, 20–39 and 40). All the four static balance tests with eyes closed were summed to create a static balance eyes closed (SBEC) variable.

Blood lead and cadmium levels were put into two categories (<5, ≥5 µg/dL for lead and <1, ≥1 µg/L for Cd). These categories were chosen in accordance with the levels of concern of the US CDC, the US Association of Occupational and Environmental Clinics and the American College of Obstetricians and Gynecologists for Pb,^33–36^ and the German Federal Environmental Agency for Cd.^37^ Blood levels were also categorised into quartiles.

The χ^2^ test was used to compare categorical variables. Unadjusted and adjusted logistic regression analyses were used to investigate the association of blood levels with balance variables.

## Results

As previously reported, the mean child blood lead level was 4.22±3.12 µg/dL (n=582)^3^ ^27^; the mean prenatal blood lead level was 3.67±1.47 µg/dL (n=4285), and the mean prenatal cadmium level was 0.58±0.63 µg/L (n=4286).^38^ ^39^ Mothers who consented to provide a blood sample were better educated and older than mothers who did not.^38^ Children who had lead levels measured were from families where the mother was better educated and more likely to be a homeowner, and there was a better home environment with fewer adversities.^3^

### Associations of measures of balance with prenatal lead and cadmium levels, and with child lead levels

#### Associations with prenatal exposure to lead and cadmium

There was no evidence of any association between the results of the heel-to-toe test at age 7 years and maternal lead or cadmium level during pregnancy (p=0.861 and 0.112, respectively) (table 1). Similarly, at age 10 years, there were no associations between dynamic balance (beam walking) or static balance (SBEC) and maternal blood lead or cadmium levels in pregnancy (all p>0.1) (table 1).

**Table 1:** Associations of prenatal lead and cadmium exposure with measures of balance in the child at 7 and 10 years of age in ALSPAC

			Maternal Pb (µg/dL)			Maternal Cd (µg/L)		
	Age (years)	Category	<5	≥5	p Value (χ^2^)	<1	≥1	p Value (χ^2^)
Heel-to-toe test	7	0–5 steps	520 (27.1)	82 (26.5)	0.861	521 (26.6)	81 (29.1)	0.112
		6–10 steps	273 (14.2)	45 (14.5)		270 (13.8)	49 (17.6)	
		11–15 steps	1128 (58.7)	183 (59.0)		1164 (59.6)	148 (53.2)	
Beam walking (dynamic balance)	10	Q1	431 (23.4)	70 (24.1)	0.450	439 (23.3)	62 (25.3)	0.897
		Q2	461 (25.0)	80 (27.6)		484 (25.6)	57 (23.3)	
		Q3	456 (24.8)	66 (22.8)		463 (24.5)	59 (24.1)	
		Q4	494 (26.8)	74 (25.5)		503 (26.6)	67 (27.3)	
Static balance eyes closed score (static balance)	10	Q1	460 (24.4)	74 (25.8)	0.558	472 (25.5)	63 (25.8)	0.842
		Q2	459 (25.4)	58 (20.2)		455 (24.6)	62 (25.4)	
		Q3	430 (23.8)	83 (28.9)		457 (24.7)	57 (23.4)	
		Q4	459 (25.4)	72 (25.1)		469 (25.3)	62 (25.4)	

Values are n (%).

ALSPAC, Avon Longitudinal Study of Parents and Children; Q, quartile.

In logistic regression models adjusted for sex, passive smoking and calcium and iron intake, there was no evidence of any association between maternal blood lead or cadmium levels, and measure of balance in the child at 7 and 10 years of age (all p>0.1) (table 2). When the models were repeated with quintiles of maternal blood lead or cadmium level rather than a dichotomous variable, there was also no evidence of any associations (all p>0.1) (see online supplementary table S1).

**Table 2:** Models of associations of in utero lead and cadmium exposure with measures of balance in the child at 7 and 10 years of age in ALSPAC

		Prenatal lead exposure				Prenatal cadmium exposure			
	Age (years)	OR of balance dysfunction (95% CI)		p Value	n	OR of balance dysfunction (95% CI)		p Value	n
Heel-to-toe test	7	Unadjusted	1.01 (0.96 to 1.07)	0.503	2231	Unadjusted	0.81 (0.70 to 0.94)	0.010	2233
		Adjusted*	1.01 (0.95 to 1.01)	0.555	1732	Adjusted*	0.95 (0.77 to 1.20)	0.904	1734
Dynamic balance	10	Unadjusted	1.01 (0.95 to 1.08)	0.790	2132	Unadjusted	1.00 (0.84 to 1.21)	0.946	2134
		Adjusted†	1.02 (0.95 to 1.09)	0.692	1761	Adjusted†	1.20 (0.95 to 1.52)	0.135	1763
Static balance	10	Unadjusted	0.98 (0.92 to 1.05)	0.569	2095	Unadjusted	1.06 (0.88 to 1.28)	0.523	2097
		Adjusted†	0.98 (0.92 to 1.06)	0.661	1734	Adjusted†	1.00 (0.79 to 1.26)	0.995	1736

Logistic regression showing OR of balance dysfunction (95% CI).

*Adjusted for: sex, passive smoking at 77 months old (weekdays and weekends), and Ca and Fe intakes at 7 years.

†Adjusted for: sex, passive smoking at 103 months old (weekdays and weekends), and Ca and Fe intakes at 10 years.

ALSPAC, Avon Longitudinal Study of Parents and Children.

#### Associations with child lead level

There was no evidence of any association between the results of the heel-to-toe test at 7 years of age and child lead level (p=0.146) (table 3). Similarly, at 10 years of age, there were no associations between dynamic balance (beam walking) or static balance (SBEC) and child blood lead levels (p=0.798 and p=0.918, respectively) (table 3). In logistic regression models adjusted for sex, passive smoking, and calcium and iron intakes, there was no evidence of any association between child blood levels at 30 months and measure of balance at 7 and 10 years of age (table 4; all p>0.3). When the models were repeated with quintiles of child blood lead rather than a dichotomous variable, there was also no evidence of any associations (all p>0.1) (see online supplementary table S1).

**Table 3:** Associations of child lead level at 30 months with measures of balance at 7 and 10 years of age in ALSPAC

			Child Pb (µg/dL)		
Test	Age (years)	Category	<5	≥5	p Value (χ^2^)
Heel-to-toe test	7	0–5 steps	82 (25.8)	34 (30.9)	0.146
		6–10 steps	52 (16.4)	22 (20.0)	
		11–15 steps	184 (57.9)	54 (49.1)	
					
Beam walking (dynamic balance)	10	Q1	74 (24.0)	24 (23.5)	0.798
		Q2	68 (22.1)	19 (18.6)	
		Q3	86 (27.9)	34 (33.3)	
		Q4	80 (26.0)	25 (24.5)	
					
Static balance eyes closed score (static balance)	10	Q1	80 (26.5)	22 (22.0)	0.918
		Q2	70 (23.2)	30 (30.0)	
		Q3	77 (25.5)	24 (24.0)	
		Q4	75 (24.8)	24 (24.0)	

Values are n (%).

ALSPAC, Avon Longitudinal Study of Parents and Children; Q, quartile.

**Table 4:** Child blood lead level at 30 months and measures of balance at 7 and 10 years of age in ALSPAC

	Age (years)	OR of balance dysfunction (95% CI)		p Value	n
Heel-to-toe test	7	Unadjusted	0.98 (0.92 to 1.04)	0.618	428
		Adjusted*	0.98 (0.92 to 1.05)	0.778	354
Dynamic balance	10	Unadjusted	1.03 (0.96 to 1.11)	0.422	410
		Adjusted†	1.01 (0.93 to 1.09)	0.814	363
Static balance	10	Unadjusted	1.04 (0.96 to 1.12)	0.345	402
		Adjusted†	1.03 (0.94 to 1.12)	0.540	357

Logistic regression analysis comparing results for children with blood lead level ≥5 vs <5 µg/dL.

*Adjusted for: sex, passive smoking at 77 months old (weekdays and weekends), and Ca and Fe intakes at 7 years.

†Adjusted for: sex, passive smoking at 103 months old (weekdays and weekends), and Ca and Fe intakes at 10 years.

ALSPAC, Avon Longitudinal Study of Parents and Children.

#### Associations with questionnaire items at 18–81 months and at 10 years

There was no evidence of any associations between child blood lead level at 30 months and the ability to stand on one foot for at least 1 s at 30, 42 or 81 months (all p>0.6; see online supplementary table S2). There was no evidence of any associations between maternal blood lead level during pregnancy and the ability of the child to stand on one foot for at least 1 s at 18, 30, 42 or 81 months (all p>0.3) (see online supplementary table S2). There was no evidence of any associations between maternal blood cadmium level during pregnancy and the ability of the child to stand on one foot for at least 1 s at 42 or 81 months (all p>0.4), but there were associations at 18 and 30 months (p<0.001 and 0.003, respectively; maternal cadmium ≥1 µg/L was associated with being more likely to be able to stand on one foot well) (see online supplementary table S2).

There was no evidence of any association of elevated child lead level, or in utero lead or cadmium exposure, with ability to stand on one leg or to walk in the dark at 10 years (all p>0.09, χ^2^ test) (see online supplementary table S3). However, prenatal lead level ≥5 µg/dL was associated with not being able to ride a bike without stabilisers (p=0.007), whereas child lead level ≥5 µg/dL and prenatal cadmium ≥1 µg/L were weakly associated with being able to ride a bike without stabilisers very well (p=0.050 and 0.075, respectively). When these associations were modelled in a logistic regression analysis adjusted for variables that could affect availability of a bicycle and being able to ride a bicycle locally (traffic level on the home street, type of accommodation, lowest level of accommodation), and maternal education, there was very weak evidence for an association of child lead level being associated with being able to ride a bike well without stabilisers (OR in unadjusted model 2.79 (95% CI 0.096 to 8.10), p=0.059, n=413; OR in adjusted model OR 2.57 (95% CI 0.87 to 7.62), p=0.089, n=387). The association was stronger for prenatal lead level in the unadjusted model, but the effect was attenuated with adjustment (OR in unadjusted model 0.59 (95% CI 0.40 to 0.87), p=0.007, n=2150; OR in an adjusted model 0.73 (95% CI 0.47 to 1.14), p=0.167, n=1904); for prenatal cadmium level, the weak effect was again attenuated by adjustment (unadjusted OR 1.69 (95% CI 0.94 to 3.01, p=0.078, n=2151; adjusted OR 1.44 (95% CI 0.75 to 2.75, p=0.274, n=1905).

## Discussion

We did not find any evidence of an association of prenatal exposure to lead or cadmium, or lead levels in childhood, on balance ability (static and dynamic) in children. Counterintuitively, there was a suggestion that higher child lead levels and in utero lead exposure were associated with the ability to ride a bike without stabilisers at age 10 years, but these effects were negated when the associations were adjusted for variables that included the lowest level of accommodation and traffic levels outside the home. This is the first study, to the best of our knowledge, reporting on the associations between in utero exposure to lead and cadmium and balance ability of the child, and adds to the few studies on child lead levels and balance ability.

Postural balance is controlled by a complex interaction of sensorimotor processes, including visual, proprioception and the vestibular system. In our study, the measure of static balance with eyes closed eliminated vision and minimised proprioceptive inputs, thereby enabling the assessment of vestibular information as the primary input. Use of clinical measures similar to these is commonplace in epidemiological studies,^40–43^ as the tests are in common clinical usage and require little or no specialist equipment. However, although the vestibular dominant condition of tests of standing balance (reduced base of support with eyes closed) has been shown to correlate well with more expensive systems such as computerised dynamic posturography (considered to be the ‘gold standard’ method for assessing balance function) in adults,^44^ there are questions about whether such a correlation exists in children.^45^ It has even been suggested that computerised measures using a force-platform and clinical tests such as those used by ALSPAC give complementary rather than concurrent information.^46^ A cautious approach should therefore be taken if seeking to compare studies using these two different types of outcome measures.

Most studies on lead levels and balance in children have included children with relatively high lead levels (means in each study of 11.6–20.7 µg/dL) and measured balance with a force platform system,^12–15^ and have found negative associations. There are several reasons for our results being in contrast with these studies. First, the mean child blood lead level in our study (4.22 µg/dL) was lower than reported in these earlier studies, and may have been too low to have been sufficient to cause balance dysfunction. Alternatively, the effect on balance might have been too small to be detectable with our tests, although a study in Inuit children had levels that were more comparable with the present study (mean 5.4 µg/dl) showed a significant association with sway oscillations.^16^ Second, we used a series of assessments based on clinical tests to measure balance rather than a force platform or measurement of sway oscillations, and this may account for differences in the findings. As discussed earlier, clinical and force platform measures may be giving complementary rather than concurrent information, whereas posturography is a measure of the motor and sensory strategies used to control balance, clinical tests evaluate the results of that balance control.^46^ Measuring sway oscillations using a force platform will also be more sensitive than the ALSPAC measures, which measured time before a procedural fault such as touching the floor with either foot or lifting a foot off the beam. Third, early exposure to lead and/or cadmium could damage the vestibular system, but the plasticity of the balance system might compensate for this so that there is no functionally measurable effect. This is in accordance with measures of balance in the present study indirectly assessing the vestibular system. This could also account for effects being reported in adults, in whom plasticity is less effective for overall balance compensation, but not in children. It is also possible that vestibular system (to include peripheral (ear) and central (brain) components of the vestibular pathway) dysfunction caused by in utero exposure to lead or cadmium may not be apparent in childhood but may manifest in later life. Finally, it is also possible that studies showing non-significant results have tended not to have been published.

To our knowledge, there are no studies that have examined the effect of in utero exposure to lead or cadmium on balance ability in the child. Our results provide preliminary evidence for lack of effect, but this requires confirmation in other cohorts.

The strengths of the study are that: (1) it is a population-based study; (2) the data were collected prospectively; and (3) the numbers included in the study were large compared with several other studies. There are several limitations to the study. First, measures of blood lead and cadmium do not necessarily reflect lifetime exposure. Bone lead, which makes up more than 95% of the body lead, can be measured by K X-ray fluorescence,^47^ but this is expensive, technically demanding, and may not always be ethically permissible in children. This limitation is of less consequence for in utero exposure, as the maternal blood level largely determines the fetal blood level. Second, there was a high proportion of blood cadmium levels below the limit of detection, which may make the results less reliable than for lead. Third, there may be confounders that we were unable to account for. Fourth, we were unable to control for lead and cadmium separately in the models because of multicollinearity. Finally, the balance measures used by ALSPAC had low test–retest reliability,^28^ which is a common problem with measures of childhood balance.^48–50^ The measure of ability to balance on one foot for 1 s, a test which was originally developed by Chamberlain and Davey^51^ in 1976, has been particularly criticised for having poor test validity, as it is difficult to discriminate a failed attempt from a successful attempt.^10^ It should ideally form part of a battery of clinical measures, perhaps with a longer duration of standing. These combined effects may have led to random misclassification in our balance assessments, and dilution of the estimates of the effects of lead and cadmium exposure.

## Conclusions

We did not find any evidence of an association of prenatal exposure to lead or cadmium with balance ability in children. In contrast with previous studies, we did not find any association of child blood lead with balance ability in children. This may reflect variation in the methods used to assess balance in different studies, or may be related to the lower mean lead level in the children in the present study than in previous studies. Further work in other cohorts is needed to be carried out to confirm the results.
